# The application of cognitive behavioral therapy in patients with schizophrenia: A review

**DOI:** 10.1097/MD.0000000000034827

**Published:** 2023-08-11

**Authors:** Feifei Xu, Hang Zhang

**Affiliations:** a School of Psychology, Zhejiang Normal University, Jin Hua, China; b School of Humanities and International Education Exchange, Anhui University of Chinese Medicine, HeFei, China.

**Keywords:** cognitive behavioral therapy, negative symptom, positive symptom, schizophrenia, social function

## Abstract

The aim of this review is to explore the clinical nursing application of cognitive behavioral therapy (CBT) in patients with schizophrenia. A literature search was conducted using the CINAHL and MEDLINE databases. The database search occurred during the month of December 2022. This article comprehensively summarizes the theoretical basis of CBT in improving schizophrenia in clinical nursing, its application in managing symptoms and improving social function, as well as research progress in this field. There are still inconsistencies in the research results on CBT, but overall, psychological intervention combined with drug treatment is more effective than conventional treatment alone. If social function training can be added at the same time, it is believed that it will have better effects on clinical treatment and can maintain long-lasting effectiveness. Only in this way can patients truly understand and recognize the disease, improve treatment compliance, and ultimately achieve the goal of improving prognosis and quality of life.

## 1. Introduction

Schizophrenia is a prevalent mental illness affecting 0.3% to 0.7% of the population throughout their lifetime. It typically manifests in young adults and is marked by disturbances in perception, cognition, emotions, and behavior, as well as a lack of coordination in mental activities,^[1]^ such as positive symptoms, negative symptoms, and cognitive impairment.^[2]^ The illness has a prolonged course and can cause a decline in mental activity, which can significantly affect the daily lives of patients. This places a heavy burden on both patients and their families.^[3]^ Antipsychotic medication is currently the most commonly used clinical treatment for schizophrenia.^[4]^ However, it is important to consider the significant side effects of such medication, as well as the stigma associated with mental illness and the negative impact of long-term medication on patient compliance^[5]^ Therefore, it is crucial to find a better treatment for schizophrenia. Research suggests that relying solely on medication therapy may not be optimal for treating schizophrenia.^[6]^ In fact, treatment with antipsychotic drugs has been linked to changes in brain volume in patients with this condition. Specifically, the volume of gray matter in schizophrenic patients tends to decrease over time, while the volume of the lateral ventricle increases. Furthermore, some of these neuroanatomical changes may be associated with the use of antipsychotic drugs.^[7]^ Ap M et al focus on patients taking olanzapine as the research object and using cognitive behavioral therapy (CBT) and conventional treatment as intervention methods, the results showed that CBT produced statistically significant improvements in total symptoms by the end of treatment. Thus, a combination of medication and psychotherapy is likely to produce better clinical outcomes for patients with schizophrenia.^[8]^

## 2. Methods

### 2.1. Aim

The aim of this review is to explore the clinical nursing application of CBT in patients with schizophrenia. This article comprehensively summarizes the theoretical basis of CBT in improving schizophrenia in clinical nursing, its application in managing symptoms and improving social function, as well as research progress in this field. In addition, this article emphasizes future research directions and potential issues that should be considered when using CBT as a treatment plan for schizophrenia.

### 2.2. Searching strategy

A literature search was conducted using the CINAHL and MEDLINE databases. The database search occurred during the month of December 2022. A search was conducted using the key terms, “Schizophrenia” AND “CBT OR Cognitive behavioral therapy.” MeSH terms were used in the MEDLINE search, and CINAHL Headings were used when searching CINAHL when available. The search limiters included the following: “English language” and “peer-reviewed.” No time limit was set.

## 3. Results

### 3.1. The therapeutic effect of CBT on different symptoms of schizophrenia

Previous research has discovered that many early-stage psychiatric patients may acquire drug resistance throughout treatment, particularly after 12 weeks of complete treatment, with approximately 20% of patients still having significant residual positive symptoms.^[9]^ The effectiveness of traditional treatments for schizophrenia is limited. However, studies have shown that CBT is a more effective long-term treatment option compared to conventional treatments.^[10]^ Rector and Beck concluded that CBT plus routine care (pharmacotherapy and case management) led to substantial pretreatment-posttreatment improvements in positive symptoms, negative symptoms, and total symptoms. Patients receiving only routine care did much less. Alternative psychosocial interventions, such as supportive therapy or befriending, produced intermediate.^[11]^ Patients who undergo CBT experience improvements in overall symptoms, positive symptoms, and social function.^[12]^ After receiving mental health services for the first time, psychiatric patients often experience a continuous period of symptom relief, with almost half of them recovering.^[13]^ The preferred method of psychotherapy is CBT. CBT helps individuals to evaluate, challenge, and change their irrational beliefs, leading to a change in their behavior.^[10]^ Initially, Aaron Beck used CBT in 1952 to treat delusional symptoms in patients with paranoid schizophrenia.^[14]^ After decades of development, CBT has made significant progress in the study of schizophrenia, with studies showing that combining CBT with medication is more effective in reducing positive psychotic symptoms than medication alone.^[15]^ CBT should be the first psychosocial intervention to be considered in the long-term treatment of patients with schizophrenia.^[16]^ However, CBT encompasses various therapeutic techniques, but different studies may use different techniques during the treatment process, due to difficulties with recruitment, the sample size is too small, resulting in inconsistent research results. This issue needs to be taken seriously in future research.^[17,18]^ Additionally, researchers may have varying levels of expertise, which can result in inconsistent findings in the study of schizophrenia. In terms of neurobiology, improvement in psychotic symptoms was predicted by neural responses to threat-related influences across sensorimotor and frontal limbic areas, whereas improvement in affective symptoms was predicted by neural responses to fearful faces only and pro-social influences across sensorimotor and frontal areas. These findings suggest that CBT is most likely to improve psychiatric and affective symptoms in those who support more threatening assessments and emotionally congruent processing biases, respectively, which are explored and redefined as part of the treatment.^[19]^ An MRI study showed that cognitive behavior therapy showed decreased activation of the inferior frontal, insula, thalamus, putamen and occipital areas to fearful and angry expressions at treatment follow-up compared with baseline.^[20]^ The purpose of this article is to explore the theoretical basis of CBT for treating schizophrenia, as well as its current research status on the symptoms and social functioning of schizophrenia. It also proposes new ideas for future research on CBT in schizophrenia.

Schizophrenia is a complex clinical syndrome characterized by a variety of symptom clusters. It is a multifactorial disease that presents with both positive and negative symptoms.^[21]^ Positive symptoms include hallucinations, delusions, excitement, bizarre behavior, and obvious thought association abnormalities.^[22]^ Negative symptoms include speechlessness, attention deficit, emotional retardation, apathy, and social withdrawal.^[23]^ With in-depth research on patients with schizophrenia, cognitive dysfunction is also an important core symptom of schizophrenia, and is a major cause of disability in schizophrenia patients,^[24]^ and it is pointed out that cognitive dysfunction appears before symptoms.^[25]^ Schizophrenia shows a variety of neurocognitive impairments, including memory, working memory, executive function, attention, and processing speed.^[26]^ Cognitive dysfunction is closely linked to symptoms and can persist even after psychotic symptoms have subsided. The improvement of cognitive function is strongly correlated with the effectiveness of treatment.^[27]^ For example, hallucinations are further related to increased suffering (evaluated as malicious, powerful, familiar voices; individuals disagree and refuse the voices), delusions, pessimistic thinking, low self-esteem, negative emotions, and cognitive impairment (executive dysfunction, tendency to draw conclusions, and inability), ultimately leading to difficulty in understanding others’ psychological states.^[28]^ Patients with severe negative symptoms will have impaired social and occupational functioning, which may cause a vicious cycle and reduce the ability of patients to learn from mistakes, ultimately exacerbating cognitive dysfunction.^[29]^ These studies indicate that cognitive function is closely related to psychotic symptoms, furthermore, after standard treatment with antipsychotic drugs, the cognitive function of patients with schizophrenia cannot be completely improved and may require adjuvant treatments.^[30]^ So, during the treatment process, not only symptoms should be treated, but corresponding treatment plans should also be taken for cognitive function improvement. Related studies have shown that CBT can improve patients’ cognitive function through cognitive regulation, thereby promoting symptom improvement.^[31]^

Research has shown that the close association between symptoms of mental illness and cognitive function has led to CBT being used as an adjunctive therapy for schizophrenia and other mental illnesses in clinical research and practice.^[32,33]^ The treatment process for positive symptoms involves thoroughly evaluating the patient symptoms,^[34]^ gradually establishing a negative relationship between cognition and experience of mental illness, questioning the evidence of automatic thinking and inference using Socratic methods,^[35]^ guiding the search for alternative explanations for voices, suspicious and delusional ideas, correcting cognitive distortions, and encouraging the adoption of more logical thinking and rational responses.^[36]^ The core principle is to challenge beliefs about content, identity, and power related to voices, with the goal of empowering patients and strengthening their self-control over their behavior (e.g., respect for voices).^[37]^ Cognitive-behavioral interventions for negative symptoms aim to change unreasonable beliefs that have a significant impact on symptoms (negative beliefs about social belonging and performance, erroneous judgments of one own ability, low expectations of happiness and success, and negative beliefs activated by positive symptoms).^[38]^ Research has shown that CBT improves psychiatric symptoms, disease prognosis, and quality of life through cognitive intervention, and the treatment effect persists for 3 months after treatment ends.^[39]^

Although the frequency and quality of cognitive intervention techniques used in CBT have different effects on symptom improvement, the overall effect is better than that of traditional therapies. However, the mechanism of action of these techniques, such as brain mechanisms, Physiological changes, is still unclear. Further analysis of the mechanism of action of CBT would be extremely important for improving the prognosis and quality of life of individuals with schizophrenia.

### 3.2. Positive symptoms

CBT can be applied as an augmentation to antipsychotic medication. The success of CBT depends on the reduction of catastrophic appraisals, thereby reducing the concurrent anxiety and distress. CBT aims at reducing the emotional distress associated with auditory hallucinations, CBT enhances personal coping strategies, enabling patients to manage symptoms and daily stress more effectively.^[40,41]^ However, in a study on auditory hallucinations in schizophrenia. A study used CBT and Cognitive Adaptation Training (CAT) - a method that provides compensatory strategies and environmental support for patients - to intervene in 2 groups of patients respectively. The results showed that the improvement of auditory hallucinations in the CBT group was not as good as that in the CAT group, and the social function improvement in the CBT group was also poorer than that in the CAT group. Compared with previous research, the difference was significant. The reason for this result may be that the average educational level of the patients recruited was too low, and they could not agree with the examiner in understanding CBT,^[12]^ indicating that the patient educational background is also a factor affecting the treatment effect.^[42]^ Moreover, delusions, a positive symptom, may be directly based on negative views of the self.

Freeman used CBT therapy to modify negative beliefs in patients with schizophrenia and found significant improvements in patients mental health, positive self-beliefs, negative social comparison, and self-esteem after treatment. Meanwhile, the degree of paranoid delusions in patients decreased significantly compared to before treatment, whereas patients in the control group who received conventional treatment did not show this improvement.^[43]^ This indicates that the effects of CBT therapy are multifaceted and can be reflected in patients’ daily lives. In order to test the replicability of the research results, the researchers conducted a comparison between 20 sessions of CBT intervention and a control group. The results showed significant decreases in scores on the Positive Symptom Scale and Delusion Scale, and in the follow-up after 12 months, the intervention effects of the CBT group were still present. This also suggests that the cognitive intervention of CBT has a significant impact on symptom improvement, and cognitive changes can maintain symptom improvement for a long time period.^[44]^

In addition, another form of psychotherapy, support therapy (ST), is also commonly used in Western clinical studies. In various studies, these 2 therapies have similarities and can both produce good results for patients. To compare the efficacy of these 2 therapies, Chinese scholars used CBT and ST in combination with medication to improve the psychopathology and insight of schizophrenic patients. In the long term, CBT was significantly superior to ST in terms of overall symptoms, positive symptoms, and social functioning of schizophrenic patients.^[45]^ This suggests that different backgrounds and populations may be the reasons for bias, ultimately leading to heterogeneous results. The above studies indicate that CBT therapy also has certain requirements for patients themselves, such as comprehension and self-control, as well as cultural background, all of which can affect the final treatment effect. Results of psychological interventions may be inconsistent in different cultural backgrounds, which suggests that in future research, treatment techniques suitable for their ethnic group should be developed based on cultural backgrounds in order to achieve the best results.

### 3.3. Negative symptoms

The characteristics of negative symptoms include emotional deficits or reductions, as well as social and behavioral impairments. They are considered one of the core symptoms of schizophrenia and are also one of the most important predictors of its quality of life and functional outcomes.^[46]^ In addition, negative symptoms may be transient or a side effect of antipsychotic medication.^[47,48]^ Treating negative symptoms of schizophrenia presents a significant challenge for mental health care. So researchers need to pay attention to drug side effects in their studies and try to adjust the number of treatments to make the intervention outweigh the drug side effects as much as possible.

A randomized controlled trial found that conducting 20 sessions of CBT within a 6-month period was effective in reducing negative symptoms in patients with schizophrenia. Additionally, improvements in cognitive abilities, daily functioning, emotional experiences, and maladaptive social beliefs indirectly led to changes in negative symptoms.^[49]^ The change in cognition promotes symptom recovery, which is also supported by relevant research in recent years. However, there is still relatively little research on negative symptoms in clinical settings, and only through large-sample randomized controlled experiments can the effectiveness of CBT be truly clarified.^[50]^ If the negative symptoms of psychotic patients are not resolved for a long time, they may be transformed into other mental illnesses such as depression,^[51]^ causing significant damage to their lives and social functioning.

Polese et al conducted a systematic analysis of studies on CBT interventions for patients with schizophrenia over the past decade and found that the efficacy of CBT in treating general psychopathological and positive symptoms was relatively clear, but its efficacy in treating negative symptoms remained unclear.^[50]^ This may be due to the lack of focus on negative symptoms in previous studies and the lower level of patient engagement in CBT interventions, which can lead to poor treatment outcomes.

However, some studies have found that group therapy can significantly increase patient engagement and have therapeutic effects. In the future, combining CBT with group therapy or social psychological interventions may improve abnormal attitudes and enhance treatment outcomes.^[52]^ Some studies have also found significant correlations between schizophrenic patients’ abnormal attitudes and beliefs, negative symptoms, and social functioning.^[53]^ CBT can target patients’ negative attitudes and beliefs centered around failure to treat their negative symptoms and functional impairments.^[9]^ Although the CBT approach is heterogeneous, numerous randomized controlled trials have found that combining CBT with standard treatments (including medication) can reduce psychiatric symptoms such as depression and negative symptoms.^[54]^

To promote the application of CBT therapy, many researchers have begun to integrate it with cognitive-behavioral social skills training, which is a comprehensive psychological intervention technique that combines CBT and problem-solving techniques to improve negative symptoms and functional impairment in patients with schizophrenia. Studies have shown that this integrated technique can achieve better therapeutic outcomes than single-drug therapy or CBT alone.^[55]^ This indicates that the use of CBT alone has limitations, whereas CBT in combination with drug therapy or other psychological treatment techniques can produce better results than conventional treatment. Future research can focus on integrating CBT with more techniques such as group therapy and social interventions to enhance the final therapeutic outcomes.

### 3.4. Social function

CBT has been proven to be a promising additive treatment method. They have been proven to improve social adaptation and quality of life, reduce symptoms of mental illness and related pain.^[56]^ Inducing and reducing apathy in social attitudes during treatment may be beneficial for patients with schizophrenia. It can also bring better social functions.^[57]^ Patients with schizophrenia face obstacles in interpersonal communication and vocational abilities, which seriously affect their social functioning and quality of life, leading to the persistence of the disease. Researchers selected early-stage patients with non-emotional mental illness for intervention by comparing CBT with conventional treatment and found that CBT can shorten hospitalization time and improve general social function. In non-emotional mental illness patients with impaired social function, CBT has important benefits and significant improvements in symptoms and emotional states.^[58]^ However, A meta-analysis suggests that CBT can have a certain effect on social function and quality of life, but its effects are not long-lasting. Improving function is a long-term goal, and drugs are difficult to play a major role, multiple joint interventions are needed to achieve better results.^[59]^

In summary, there are still inconsistencies in the research results on CBT, but overall, psychological intervention combined with drug treatment is more effective than conventional treatment alone. If social function training can be added at the same time, it is believed that it will have better effects on clinical treatment and can maintain long-lasting effectiveness. Only in this way can patients truly understand and recognize the disease, improve treatment compliance, and ultimately achieve the goal of improving prognosis and quality of life.

## 4. Future research directions

A study consisting of 36 randomized controlled trials involving 3542 patients with schizophrenia compared and analyzed various indicators such as relapse, readmission, mental state, mortality, social function, and quality of life between CBT and other psychosocial therapies. The results showed no significant differences between CBT and other therapies, and the effectiveness of CBT could not be conclusively determined. This could be attributed to flaws in experimental design.^[60]^ The clinical efficacy of CBT is commendable, however, schizophrenia is still one of the main causes of disability worldwide. Current research on CBT interventions in schizophrenia mostly focus on symptomatic recovery, that is, on mental functions, as a primary treatment target.^[61]^ Although symptom reduction is undeniably important in schizophrenia it is less clear the extent to which traditional and “third wave” CBT interventions address the whole scope of disabilities experienced by people with lived experience of schizophrenia. Considering service users definitions of recovery it is also important to explore, whether current CBT interventions focus on recovery and what is their impact on disability domains.

In the future, we can start from the treatment plan and find a rigorous research approach to ensure the consistency of research results and provide more evidence for the clinical application of CBT.

Such results may be due to CBT being a commonly used adjunctive therapy in clinical settings. If a safe and stable therapeutic alliance cannot be formed with patients during treatment, it may result in poor patient compliance, leading to inadequate treatment efficacy. Therefore, prior to the study, sufficient contact should be made with the patients so that they can have a full understanding of the therapy, followed by further intervention. CBT primarily relies on the skills of the main examiner, and different interventions by different examiners may lead to different results for patients. Therefore, rigorous CBT training for examiners is necessary, followed by strictly following the intervention plan to intervene with patients. In the future, standardized treatment in research has great potential for clinical studies. Personalized CBT techniques should be used for different types of schizophrenia patients to achieve consistent effects. Future studies can try to study patients with a single subtype of schizophrenia and combine brain electrical activity and imaging techniques to explore the mechanism of action of CBT, providing more beneficial evidence for the joint use of computer technology or behavioral training methods in the future.

At present, there is a significant gap in both professional psychological interventionists and effective intervention plans in China. Although there are a large number of psychiatrists in China, there are still very few medical workers specialized in psychological therapy for the treatment of schizophrenia. So introducing effective interventions from abroad has important practical significance. CBT therapy has been widely applied abroad, but it is still relatively less involved in domestic research, and there is still a significant gap between domestic researchers and professionalization in treatment technology. On the one hand, referring to existing therapeutic research in the West and combining it with domestic culture, we have developed CBT technology that belongs to our own country, cultivating more psychotherapy talents. On the other hand, in the future, existing intervention plans can be improved as much as possible from the perspectives of curriculum design, content arrangement, etc, in order to strengthen the intervention effect and increase patient participation, so as to maximize the effectiveness of CBT. Internationally, CBT has achieved good clinical results, but there is still a need to pay attention to the issue of combining multiple technologies for intervention based on individual differences. At the same time, traditional CBT interventions address more disability domains than “third wave” therapies, however, both approaches focus mostly on mental functions that reflect schizophrenia psychopathology there are also few interventions that focus on recovery. These results indicate that CBT interventions going beyond symptom reduction are still needed. Recovery-focused CBT interventions seem to be a promising treatment approach as they target disability from a broader perspective including activity and participation domains.

## Author contributions

**Conceptualization:** Feifei Xu, Hang Zhang.

**Data curation:** Hang Zhang.

**Formal analysis:** Hang Zhang.

**Funding acquisition:** Feifei Xu.

**Project administration:** Feifei Xu, Hang Zhang.

**Resources:** Feifei Xu.

**Supervision:** Hang Zhang.

**Visualization:** Feifei Xu, Hang Zhang.

**Writing – original draft:** Feifei Xu, Hang Zhang.

**Writing – review & editing:** Feifei Xu, Hang Zhang.
